# Investing in stockpeople is an investment in animal welfare and agricultural sustainability

**DOI:** 10.1093/af/vfy015

**Published:** 2018-06-23

**Authors:** Courtney L Daigle, Emily E Ridge

**Affiliations:** Animal Behavior and Welfare Laboratory, Department of Animal Science, Texas A&M University, College Station, TX

**Keywords:** animal welfare, husbandry education, stockperson, sustainability, workplace psychology

**Implications**

- Stockmanship is an occupation that requires expertise, endurance, and empathy. Stockpeople have a direct impact on agricultural animal welfare and productivity. Unfortunately, stockpeople are undervalued and their role in agricultural sustainability overlooked.
- A shortage exists for good stockpeople, and investigations are needed that examine the impact of higher stockperson salaries and education on employee behavior, employee retention, applicant pools, animal welfare, and sustainability.
- Integration of husbandry education into university curricula, conducting personality assessments on potential employees, and promoting occupational awareness may facilitate the development of a long-term highly skilled stockperson workforce.

## Introduction

Stockpeople are the stewards of our food animals and play a critical role in agricultural sustainability. Yet, there is a disconnect between the value that is placed upon their role in animal agriculture, their compensation, and the scope of impact they can have on the animal’s productivity, public perception of animal agriculture, and animal welfare (Hemsworth and Coleman, 2011) in the United States and abroad (Table 1). The daily care, long-term health, and productivity of our food animals are the responsibility of the stockperson. Stockpeople are usually regarded as itinerant and unskilled which can result in managers making minimal investments into their development as employees, and producers are increasingly reporting difficulties in identifying qualified applicants. However, because of the critical role the stockperson plays in animal production and welfare, the human resources devoted to selecting, training, and managing these employees need to reflect modern employee management found in other occupations (Hemsworth and Coleman, 2011). Stockmanship is a representation of animal welfare itself, where the entirety of the animal, its physiological, behavioral, and emotional state is managed by their human caretaker, and thus requires a complex and deep skill set to properly implement.

**Table 1.** Median stockperson salaries, poverty lines, and minimum wage annual incomes for the United States, Canada, and England

	United States^1^	Canada^2^	England^3^
Stockperson annual salary	$25,470	$32,810	$29,568
National poverty line (four-person household)	$24,848	$40,295	$29,370
Minimum wage annual salary	$15,130	$20,846	$23,014

All values are in US$.

^1^Information from the Bureau of Labor Statistics. Annual median salary is from 2017 data for Farmworkers, farm, ranch, and aquacultural animals. Poverty line and minimum wage annual salary are 2018 data.

^2^Information from Statistics Canada: Canada’s National Statistical Agency. Stockperson salary is for occupation NOC 8252—Agricultural service contractors, farm supervisors, and specialized livestock workers. National poverty line is the 2017 Low Income Measure.

^3^Information from www.gov.uk. Stockperson salary is calculated as a grade 6 Agricultural Worker that works 6 d per week.

As described by Hemsworth and Coleman (2011), the stockperson is required to have:

a basic knowledge of both the behavior of the animal and its nutritional, climatic, housing, health, social and sexual requirements together with a range of well-developed husbandry and management skills to effectively care and manage farm animals. For instance, farm personnel may have knowledge and skills in a number of diverse management and husbandry tasks such as estrus detection and mating assistance; semen collection, semen preparation and artificial insemination; pregnancy diagnosis with ultra-sonography; artificial rearing of early weaned animals; milk harvesting; controlling and monitoring of feed intake to optimize growth, body composition, milk production and reproductive performance; pasture management to optimize pasture production; routine health checks; monitoring and adjusting climatic conditions in indoor units; administering antibiotics and vaccines; shearing and crutching of sheep, teeth and tail clipping of pigs; castration of males; and effective and safe animal handling. These are skilled tasks and farm personnel are required to be competent in many of these tasks.

Substantial resources have been invested to enhance the genetic merit of livestock, maximize their reproductive capacity, and optimize the quality of the nutrition they receive. Husbandry guidelines and policies have been developed and legislation passed with the intent of promoting good animal welfare; however, the primary emphases of these initiatives are on housing requirements and pain management. The regulation of animal care has focused primarily on how animals are housed and by what means they are managed. What is sometimes forgotten is that the individual often responsible for implementing such changes is the stockperson. Therefore, the impacts from these efforts may have substantial positive, or negative, consequences depending upon the stockperson’s actions.

Investing in high-quality, well-trained, and appropriately compensated stockpeople must become a national priority within the food industry. However, a disconnect exists between stockperson pay, the level of knowledge and skill required to perform the job, and the impact these employees can have on the overall productivity and welfare of the animal (Hemsworth and Coleman, 2011). The duration of time stockpeople spend interacting with animals is substantially longer and requires more knowledge and skill than the interactions they have with the truck driver that delivers the animals to the slaughterhouse, yet the truck driver is paid almost twice as much as the stockperson (Table 2).

**Table 2.** Annual salaries from the 2017 U.S. Bureau of Labor Statistics for people who interact with agricultural animals

Occupation	Median	Percentile 10%	Percentile 90%
Farmworkers, farm, ranch, and aquacultural animals	$25,470	$18,330	$40,080
Veterinary assistants and laboratory animal caretakers	$26,140	$19,110	$38,300
Slaughterers and meat packers	$27,530	$20,330	$37,150
Construction laborers	$34,530	$22,280	$63,400
Heavy and tractor-trailer truck drivers	$42,480	$27,510	$64,000

**Figure 1.**
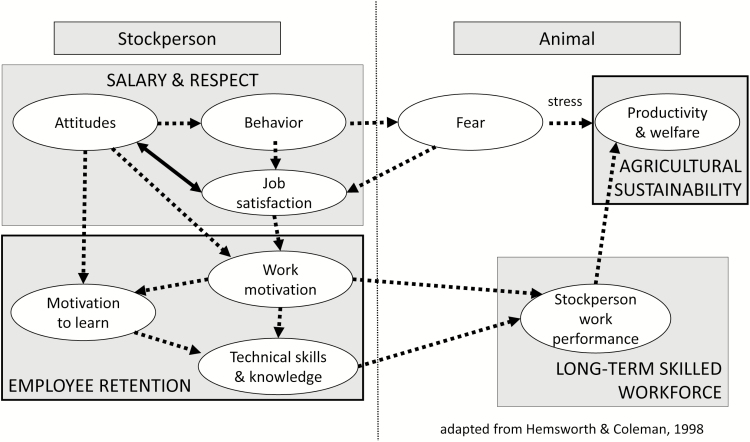
The human–animal interaction in relation to stockperson salary, job retention, and sustainability.

In concert with the change in society’s expectations regarding animal welfare, these societal expectations must expand to acknowledge stockpeople as professionals, and they must be treated as such. The American Society of Animal Science identified Training the Future Workforce as one of their Grand Challenges in 2015, particularly since the demographic of the Animal Science student differs greatly from that of the Animal Science faculty member. A need for more animal scientists exists across all disciplines, including stockmanship—which requires skill, knowledge, and training similar to other animal science disciplines. Awarding stockpeople the knowledge and respect they deserve, and incorporating stockmanship skill development and training into the undergraduate curriculum, will aid in creating a long-term, satisfied, skilled workforce. Investing in stockpeople can improve workforce morale and have positive impacts on productivity, animal welfare on farm, and has the potential to influence the overall sustainability of animal agriculture (Figure 1).

## Challenges in Developing and Retaining a Highly Skilled Workforce of Stockpeople

Society is increasingly interested in the welfare of animals that provide us with meat, eggs, and milk, but are not consistent in their willingness to pay a premium for products that are accompanied with assurances of good animal welfare (Harper and Henson, 2001; Schroder and McEachern, 2004; Carlsson et al., 2007). This creates a challenging dilemma because while consumers will express their desire for animal products that are “animal welfare friendly”, they are unwilling to financially support their ethical choices. This discourse presents an additional challenge to investing in the stockpeople that are responsible for implementing these socially valued humane and sustainable husbandry practices because while the need exists, the resources to support this need are minimal.

Emotional valence and stockperson attitude can influence stockperson behavior toward agricultural animals (Hemsworth and Coleman, 2011). Challenges that stockpeople face outside of the workplace may influence their performance in the workplace which can impact the animals in their care. Stockpeople are required to make quality of life decisions for agricultural animals on a daily basis, yet some of them may be more interested in preserving their own quality of life due to personal pressures associated with living in a low-income family. Therefore, they may be faced with difficult decisions that place their own welfare against those of the animals in their care (i.e., being paid by the head instead of by the hour, bonus compensations based upon number of pigs weaned per sow per year may cause an increase in health issues in the finishing sectors). This dynamic may be a contributing factor to the high turnover rates found in the agricultural workforce.

Low pay, increasing urbanization, the prospect of physical labor, and unrealistic expectations regarding work duties are factors that may contribute to the small applicant pool and high turnover rates of stockpeople. Turnover rates for stockperson positions in Australian swine operations have been reported to be around 50% over a 6-mo period and have been anecdotally reported to be 60% in U.S. laying hen facilities (Benson and Rollin, 2008). A continuous change in personnel can have direct and indirect impacts on animal welfare. New stockpeople must undergo a training period where, even if they have previous experience working with livestock, they must become familiar with the animals in their care, and they will need to learn the protocols and expectations associated with their new work environment. Many new stockpeople have limited animal handling experience, and therefore they will also be undergoing a period of skill development because much of their training and skill development will occur on the job. Changes in personnel require knowledge transfer, and miscommunications can result in a loss of knowledge that can have animal welfare implications (e.g., specific animal temperament, medical and treatment records, health and behavioral history of the animal, facility requirements). Further, when there is a personnel change there is also a change in the human–animal relationship, which may negatively, or positively, impact production and welfare. Implementing strategies that focus on enhancing employee retention and stockperson welfare (e.g., education, training, on-site cafeterias, convenience stores, and laundromats) may indirectly enhance animal welfare and productivity.

Employee retention is a challenge for many producers across all animal product sectors. Despite the issues associated with maintaining long-term employees and their subsequent low financial compensation, animal industry employees enjoy their work. Historical survey data demonstrated that 74% of farm workers were satisfied with their work and job satisfaction, and this enjoyment was increased with the implementation of new technologies (Ivanov and Patrushev, 1976). A majority of Australian swine and dairy stockpeople expressed a strong enjoyment for working with animals on the daily basis. (Hemsworth and Coleman, 2011). These survey data correlate to the unpublished data recently collected by the authors, who observed high levels of job satisfaction, job enjoyment, and enjoyment of working with animals in beef cattle feedyard workers in Texas.

**Figure 2.**
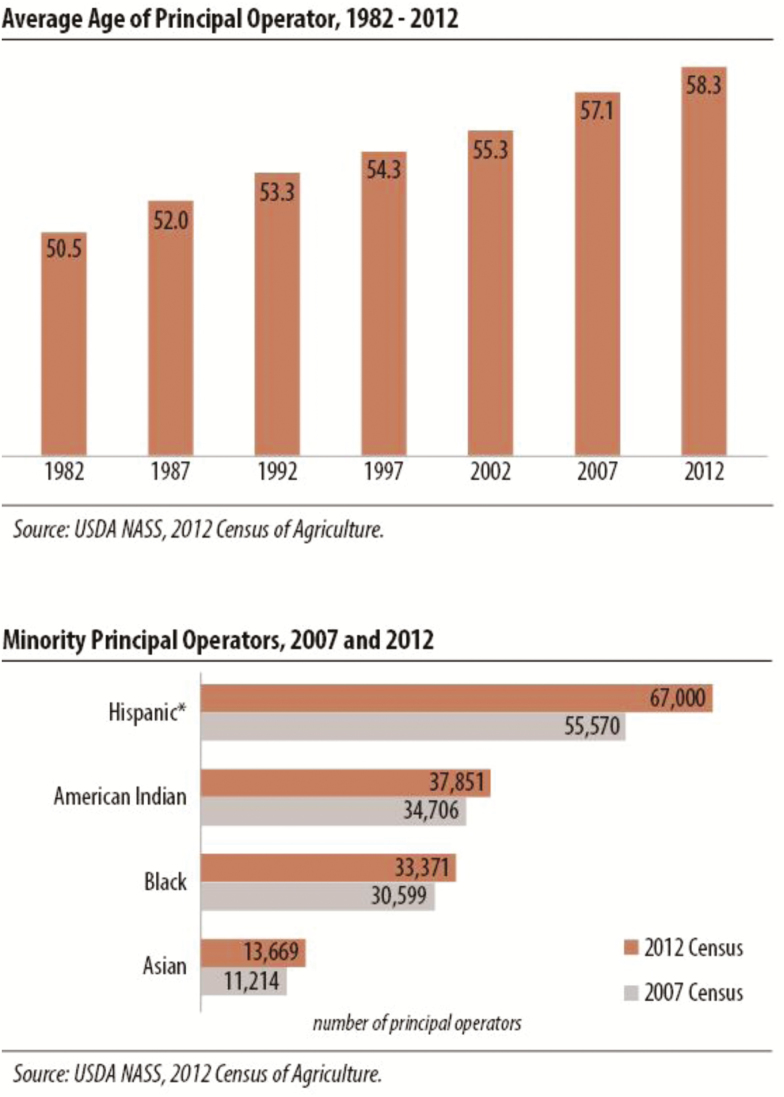
Change of average wage of animal agricultural workers in the United State (NASS, 2012).

The urbanization of the United States has resulted in fewer people working in agriculture, with the greatest decline observed in females. Between 2007 and 2012, there was a 5.9% decline in the number of female farmers while there was a 4.1% decline in male farmers. Figure 2 illustrates that the average age of U.S. farmers has steadily increased from 1982 to 2012 (NASS, 2014) and is becoming increasingly diverse with the greatest increase from 2007 to 2012 observed in Hispanic-operated farms (NASS, 2014). The shift in the agricultural population to be increasingly older and male is accompanied by a 23.3% reduction from 2007 to 2012 in the number of new farmers that have been on their current operation for less than 5 yr. This highlights that there are fewer younger people engaging in agriculture, and subsequently there are fewer potential employees available for the applicant pool.

This is an ominous trend that may have unforeseen consequences (e.g., loss of husbandry knowledge, not enough stockpeople to care for livestock) to the future of our food supply. The decline in female engagement in agriculture is particularly concerning since female stockpeople are rated more conscientious by their supervisors, and rated themselves as having greater technical knowledge and a better work ethic than males (Coleman, 2008). One strategy that may attract more people to the occupation may be providing salaries and benefits that are competitive with salaries that offer the same standard of living as an urban occupation requiring the same level of knowledge and skill. Another strategy is to increase awareness of employment opportunities and to empower those from urban backgrounds with little inherent knowledge and experience to engage with agricultural animals and to utilize their skill set in new and unique ways.

The enjoyment of working with animals combined with the positive impact that technological advancements have on worker satisfaction present a promising opportunity for future stockpeople in animal agriculture. The onset of precision agriculture and the implementation of new technologies within the animal agricultural industry will provide ample opportunities for stockpeople to engage in animals while utilizing cutting-edge technologies. Therefore, there is a need to identify individuals that are highly motivated to work with animals and are also technologically savvy. Fortunately, many of the individuals that may become stockpeople will most likely be from urban, and highly technological, backgrounds. Because precision agriculture is the future of food animal production, this suggests that those who choose to enter into this line of work will find themselves highly satisfied with what they do for as long as they choose to do so.

## Increasing Occupational Awareness and Formal Stockmanship Education

**Figure 3.**
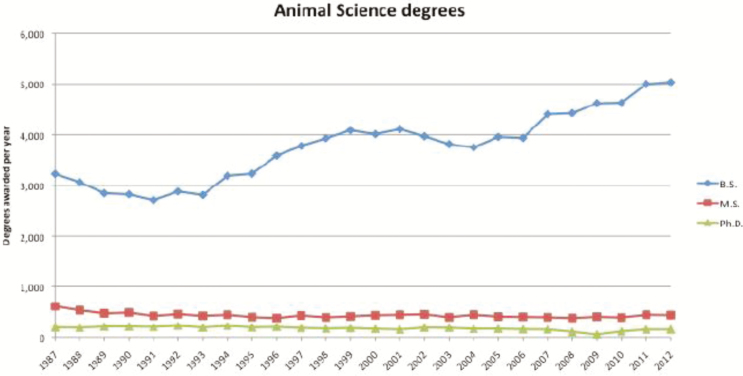
Number of BS, MS, and PhD degrees awarded over a 25-yr period (Knapp, 2014).

Investment in stockpeople is needed not just at the compensatory, but in the societal level, and there is a need to provide educational opportunities that specifically address their occupational requirements. Previous generations of agricultural workers gained much of their knowledge regarding animal husbandry during their childhood, as many Americans lived in agriculturally oriented communities. The value of this inherent knowledge and skill development has historically been overlooked as animal husbandry and management skills were not unique pieces of information. However, the U.S. population is becoming increasingly urban with more people moving to large cities and more urban areas becoming developed as a result of population increases (United States Census, U.S. Department of Commerce). Therefore, many students will begin their collegiate career without the fundamental knowledge required to care for, handle, and manage livestock. More Americans are attending 4-yr universities, and the number of Animal Science undergraduate degrees has steadily increased from 1987 to 2012 (Figure 3). The value of teaching animal husbandry is increasing because there are fewer people born in the United States that will have the opportunity to learn and possess these skills prior to beginning their undergraduate career.

The Animal Science undergraduate curriculum is continually revised and updated to include the most current knowledge regarding modern technology and advancements in Animal Science. As our knowledge has grown, so has the need to incorporate genetic, nutritional, reproductive, and physiological information in the undergraduate curriculum. Subsequently, the emphasis on teaching animal husbandry has diminished even though the need to disseminate this knowledge has increased. Many urban Animal Science undergraduates lack the inherent husbandry knowledge and animal handling skills associated with previous generations of Animal Science students. Therefore, students may graduate with an undergraduate Animal Science degree having a solid scientific understanding of how and why the animal functions, but may have limited practical knowledge regarding how to care for and manage livestock populations. Integrating animal husbandry skill development and practical management strategies into the undergraduate curriculum may boost application pools for stockmanship positions and catalyze the transformation of society’s perception of stockmanship from being considered a job for unskilled workers to a profession that requires skill, education, and respect.

## Workplace Psychology, Stockmanship, and Animal Welfare

Maintenance of animal welfare happens at the ground level beginning with the interaction between the animal and the stockperson. Curating a positive human–animal relationship is essential for the stockperson in order to ensure they are properly caring for the animals they interact with a daily basis. Icek Ajzen’s Theory of Planned Behavior states that humans act in favorable ways toward things they like and respect (Ajzen, 1991). This theory can be applied to stockperson behaviors and animal treatment based on level of satisfaction they have for their job and the animals in their care. The productivity and health of food animals have been directly connected to the attitudes and actions of the stockperson.

It is well known that stockperson behavior can elicit a fear response in livestock (Coleman et al., 1998). Fear is the perception of actual danger, and activation of the fear response has deleterious consequences on productivity and welfare. Therefore, if animals perceive a situation or human as a predator or threat, then the fight or flight response will be activated resulting in a cascade of physiological responses that detract from productivity and compromise welfare. For example, poor stockperson attitude leads to increased electric prod use in pig abattoirs (Coleman et al., 2003) and negative behaviors impacted milk yield on commercial dairy farms (Breuer et al., 2000). In order to promote sustainability through positive animal welfare and productivity, industry employers must maintain positive stockperson attitudes by ensuring their well-being in the workplace.

Animal agricultural operations can look to studies in occupational psychology that have thoroughly evaluated the needed factors to achieve job satisfaction and employee well-being. Positive emotional workplace culture, employee participation, and individual sense of belonging are all interdependent factors that promote employee well-being. Positive affection, which can be displayed in the form of respect, has been shown to encourage the pursuance of work objectives, leading to improved job satisfaction (Biggio and Cortese, 2013). Promoting self-confidence in the workplace not only has shown to benefit the individual, but also the entire morale of the workplace (Biggio and Cortese, 2013). These psychological findings are applicable to all workplaces, including those in the animal agriculture industry. Investing in employee morale on farm not only increases the individual stockperson well-being and job satisfaction but can strengthen the whole workforce.

While investing in workplace morale may increase job satisfaction and attitudes, employing different training methods on farm may improve and maintain positive stockperson behavior (Coleman and Hemsworth, 2014). A variety of studies have shown that negative behaviors toward animals from stockpeople cause an increase in avoidance behaviors and decreased productivity in many species of livestock animals (Hemsworth and Coleman, 2011). These negative behaviors have been shown to be avoided upon providing cognitive behavior training to stockpeople (Hemsworth et al., 1994). Not only does this training target their underlying beliefs of animals but also changes physical behaviors through breaking poor habits, addressing aggressive defensiveness toward animals and providing re-enforcement and follow-up of positive behaviors. This training can be coupled with increasing understanding of animal behavior to improve the overall attitudes and behaviors of stockpeople (Coleman and Hemsworth, 2014). Hemsworth and colleagues (1994) showed that by implementing cognitive behavioral training on-site at a commercial swine facility, they were able to decrease the performance of negative stockperson behaviors toward pigs while increasing employee attitudes toward pigs. By engaging stockpeople in training and providing them a positive, respectful work environment, we can improve the attitudes and behaviors of these individuals in a way that can significantly contribute to improving animal productivity and welfare. Training may also have the ability to increase self-esteem of the stockperson (Hemsworth and Coleman, 2011) which can have long-term positive ramifications for productivity and animal welfare.

## The Stockpersons Contribution to Agricultural Sustainability

Stockpeople are given substantial power and responsibility with regard to the productivity of agricultural animals. Stockperson attitudes, behaviors, and decision-making skills can either improve or compromise animal welfare, which impacts animal productivity, profitability, and social acceptability of agriculture. The number of applicants to select from is small and much of the training these employees receive is on-the-job where husbandry practices are usually implemented based upon historical knowledge and individual experience. However, individuals that wield the power to influence farm-level productivity should be specifically trained and selectively chosen to fill these positions. Thus, it is essential that animal agriculture prioritize stockperson education and training.

This is a complex problem that requires systematic and objective evaluation of the social, economic, human resources, animal welfare, and animal productivity factors that may be impacted by salary increases or additional benefits, and whether these changes that are designed to impact the stockperson can be detected in the productivity of the animals in their care. Society’s perception and value of stockpeople must undergo a similar revolution that has been observed in zookeepers (Daigle, 2016). Although employee turnover is a challenge for zoos, zookeeping has become a desirable and competitive occupation (Crosby, 2001). Within the last 20 yr, the minimum requirements for becoming a zookeeper have risen drastically where now the typical zookeeper holds a Bachelor’s degree or higher (Bunderson and Thompson, 2009).

The sense of moral duty to engage in an occupation that offers low wages and hard work is reflected in the increased efforts in zoo research, zoo animal training, environmental enrichment, assisted reproductive technology, specialized animal husbandry techniques, and conservation efforts and applicant pools worldwide (Allen, 2015). Yet, many of the fundamental requirements of the job and the salaries of zookeepers have not changed (Allen, 2015). Edward O. Wilson (1984) introduced the concept of “biophilia” in 1984 that describes “the connections that human being subconsciously seek with the rest of life.” Many humans have a strong biophilic drive and will seek opportunities to engage with animals. However, many North Americans are unaware that stockmanship is an occupation that can fulfill that desire, and this knowledge gap may contribute to the increased interest in zookeeping and decreased interest in animal agriculture as an occupation.

Caring for and working with the animals we eat should be an honorable and desirable occupation. Employment as a stockperson is a privilege and opportunity to make meaningful contributions to agricultural animal welfare, and those engaged should be motivated to lead by example. Zookeeping may be a “calling”, but the urbanite’s attraction to working with animals can, and should, be extended to agricultural animal care as well. By increasing awareness of what opportunities are available, clearly defining expectations, emphasizing the importance and impact of this occupation, and providing the education required to meet those expectations, we may be able to secure a long-term skilled workforce of stockpeople that can promote animal welfare and overall agricultural sustainability.

## About the Authors

**Courtney Daigle** is an Assistant Professor of Animal Welfare in the Department of Animal Science at Texas A&M University. She conducts research designed to quantify animal behavior and objectively evaluate management practices that enhance animal welfare. She teaches undergraduate and graduate courses and mentors graduate students in animal sciences and the human–animal interaction.

**Emily Ridge** is a graduate student in the Department of Animal Science at Texas A&M University under the auspice of Dr. Courtney Daigle. She is currently investigating the relationships between nonnutritive oral behaviors and rumen physiology in feedlot cattle. She is also investigating the attitudes and perceptions of feedyard employees.
